# Engineering saline-alkali-tolerant apple rootstock by knocking down *MdGH3* genes in M9-T337

**DOI:** 10.1007/s44154-025-00236-7

**Published:** 2025-06-23

**Authors:** Fang Zhi, Tianle Fan, Jia Li, Shuo Zhang, Qian Qian, Arij Khalil, Chundong Niu, Kun Wang, Fengwang Ma, Xuewei Li, Qingmei Guan

**Affiliations:** State Key Laboratory for Crop Stress Resistance and High-Efficiency Production /Shaanxi Key Laboratory of Apple, College of Horticulture, Northwest A&F University, Yangling, Shaanxi 712100 China

**Keywords:** Apple, Saline-alkali stress, M9-T337, *MdGH3* RNAi, Rootstock

## Abstract

Soil salinization and alkalization have become an increasingly severe global issues, significantly limiting both the yield and quality of apples (*Malus* × *domestica*). M9-T337 is a widely used apple dwarfing rootstock; however, it is sensitive to saline-alkali stress. Therefore, developing saline-alkali tolerant apple rootstocks is essential. In this study, we utilized RNAi (RNA interference) technology to knock down *GH3* genes in the M9-T337 background, aiming to engineer a dwarfing and stress-tolerant apple rootstock. We found that *MdGH3* RNAi plants exhibited superior morphology compared to M9-T337 under saline-alkali stress conditions, characterized by more robust root systems, increased plant height, a lower Na^+^/K^+^ ratio, and enhanced photosynthetic and antioxidant capacities. Moreover, when *MdGH3* RNAi plants were used as rootstocks, the GL-3/*MdGH3* RNAi plants also displayed greater plant height, root vitality, photosynthetic ability, and antioxidant capacity compared to GL-3 grafted onto M9-T337 rootstock. Taken together, our study constructed a saline-alkali-tolerant apple rootstock by knocking down *MdGH3* genes.

**Supplementary Information:**

The online version contains supplementary material available at 10.1007/s44154-025-00236-7.

## Introduction

Soil salinization and alkalization have been becoming more and more challenging, affecting over 900 million hectares in more than 100 countries, with annual losses of $27.3 billion (FAOSTAT, http://www.fao.org/faostat/). Saline-alkali stress is a complex form of stress, in which both salt and alkali are present simultaneously in the soil environment, affecting plant growth and productivity. It usually causes increased Na^+^/K^+^ ratio, severe oxidative damage and inhibited photosynthesis in plants (Ma et al. 2019; Luo et al. 2021). As one of the most widely cultivated fruits globally, apple (*Malus* × *domestica*) trees are cultivated all over the world (Liu et al. 2023). However, some apple production regions face the challenge of soil salinization and alkalization. Therefore, improving apple adaptability to saline-alkali environments has become an urgent and critical task.

Apple propagation mainly relies on grafting. Grafting is an ancient asexual propagation technique that has been widely applied in fruit trees for centuries (Goldschmidt 2014). The main purpose of grafting is to shorten the juvenile period and promote early flowering and fruiting of fruit trees (Mitani et al. 2008; Habibi et al. 2022). In the process of grafting, rootstocks play key roles in conferring plants stress tolerance to pathogens, low temperatures, salinity, and drought stresses (Cuartero et al. 2006; Zhu et al. 2014; Riga 2015; Jiang et al. 2022a, b). Currently, M9-T337 is the most widely used dwarfing apple rootstock globally, has been selected from the virus-free M9 variety developed at the East Malling Research Station in the UK (Wang et al. 2019). M9-T337 exhibits several advantages, including dwarfing, graft compatibility, early fruiting, high fruit quality, and high yield. However, research has shown that M9-T337 has poor tolerance to saline-alkali stress, which limits its application in saline-alkali soils (Tabakov et al. 2016; Zhang et al. 2023a, b; Xian et al. 2024).

Genetically modified technology is an effective method to enhance crop tolerance to saline-alkali stress. Nowadays, various genes have been applied to improve plant adaptation to saline-alkali environments. The genes that function to improve the developed root system under salt stress conditions are usually regarded as positive regulators (Li et al. 2023). Previous studies have found that the overexpression of aquaporin protein *ThPIP2;5* in *Tamarix hispida*, *GsGSTU13* in *Medicago sativa*, and *GSO1/SGN3* in *Arabidopsis thaliana* improves root length and vitality, thereby promoting Na^+^ efflux in roots and enhancing plants’ tolerance to salt stress (Jia et al. 2016; Wang et al. 2018; Chen et al. 2023). Additionally, considering the important roles of SOS signaling pathway in plants salt stress response, applying the genes involved in SOS module complex is a powerful method to modify plants tolerance to salt stress condition. For example, transgenic tobacco (*Nicotiana tabacum*) plants overexpressing *OsSOS1* show enhanced salinity stress tolerance (Gupta et al. 2021). Overexpressing the membrane-attached *SOS2* also improves Arabidopsis tolerance to salt stress (Lou et al. 2020). The Ca^2+^ sensor *CBL8* is a component that can specifically decode high-salt-stress-induced Ca^2+^ signals involved in SOS pathway. Overexpression of *CBL8* in tobacco and Arabidopsis enhances salt tolerance and root Na^+^ efflux (Steinhorst et al. 2022). In poplar (*Populus alba* × *P. berolinensis*), *JERF36s* overexpression increases *NHX1* and *SOS1* activities, maintaining plants’ intracellular Na^+^/K^+^ balance and improving salt tolerance (Ding et al. 2020). Apart from the ion balance, certain genes enhance plant adaptation to saline-alkali conditions by regulating photosynthesis, including *OsCSLD4* in rice (*Oryza sativa*) and *PagGRXC9* in poplar (Liu et al. 2024; Wang et al. 2024). Moreover, plants adapt to the saline-alkali stress conditions by enhancing antioxidant activities and hormonal balance (Qin et al. 2019; Zhao et al. 2020; Zhang et al. 2023a, b).

In plants, GRETCHEN HAGEN 3 (GH3) proteins function as acyl acid amido synthetases, catalyzing the conjugation of amino acids to IAA (Jez 2022). These proteins play crucial roles in plant development and abiotic stress responses (Luo et al. 2023). The first *GH3* gene was identified from soybeans (*Glycine max*) (Staswick et al. 2005). Previous studies have found various roles of *GH3* members in response to drought stress. Overexpression of rice *OsGH3-2* and potato (*Solanum tuberosum*) *StGH3.3* inhibits plants root development and ROS (Reactive Oxygen Species) scavenging, thereby decreasing its drought tolerance (Du et al. 2012; Yao et al. 2023). Similar roles are also found in apple *MdGH3.6* and grape (*Vitis vinifera*) *VvGH3-9* (Jiang et al. 2022b; Lu et al. 2022). However, some *GH3* genes play positive roles in plant response to drought stress. Silencing of *MdGH3-2/12* in apple and *GhGH3.5* in cotton (*Gossypium hirsutum*) reduces plants drought tolerance with accumulated ROS content and reduced photosynthesis (Kirungu et al. 2019; Huang et al. 2021). Since the root development mediated by auxin homeostasis is mainly dependent upon the function of *GH3* (Xie et al. 2022; Ke et al. 2024), current studies focus on revealing the mechanism by which *GH3* gene regulates plant response to drought stress. Few studies have focused on *GH3* genes in saline-alkali stress. Only *GH3* in *Physcomitrium patens*, *GH3.5/3.9* in Arabidopsis, and *GH3.*5 in cotton have been found to be involved in regulating plants’ response to salt stress (Han et al. 2019; Kirungu et al. 2019; Koochak and Ludwig-Müller 2021). Previously, we identified *MdGH3.6* as a target of MdMYB94 and negatively regulated apple drought stress response (Jiang et al. 2022a, b). The enhanced drought tolerance of *MdGH3* RNAi plants (*GH3.6* and its five paralogues were silenced) coffered its potential as apple rootstocks to promote the flowering, fruiting, and drought tolerance of the scion (Jiang et al. 2022a, b). However, the biological function of apple *MdGH3* in saline-alkali stress remains unknown. That might limit the application potential of *MdGH3* RNAi plants in complicated stress conditions.

In this study, we tried to knock down *MdGH3.6* and its five paralogues in the M9-T337 background to engineer a dwarfing and saline-alkali tolerant apple rootstock. The *MdGH3* RNAi transgenic apple seedlings showed the consistent morphology with the wild type M9-T337 under normal conditions, but exhibited enhanced tolerance under saline-alkali stress conditions. The more developed root system and reduced Na^**+**^**/**K^**+**^ ratio confer the tolerance of* MdGH3* RNAi plants to saline-alkali stress by improving their photosynthetic capacity and antioxidant capacity. Furthermore, the *MdGH3* RNAi transgenic apple could be used as a good rootstock with enhanced tolerance to saline-alkali stress compared to M9-T337.

## Results

### Knockdown of *MdGH3* genes improves apple tolerance to saline-alkali stress conditions

Previously, we found the potential application of *MdGH3* transgenic apple plants (in the background of GL-3) as a rootstock to enhance apple drought tolerance without the penalty of flowering, fruit setting, fruit quality, and yield. However, the *MdGH3* RNAi plants were taller than the commercial rootstock M9-T377 (Jiang et al. 2022a and b). To obtain dwarfing and stress tolerance rootstock, we transformed *MdGH3*-RNAi vector into the M9-T337 background (Fig. S1A). qRT-PCR assay found the mRNA levels of six *MdGH3s* in *MdGH3* knockdown lines decreased compared to the wild type M9-T337 (Fig. S1B-G).

**Fig. 1:**
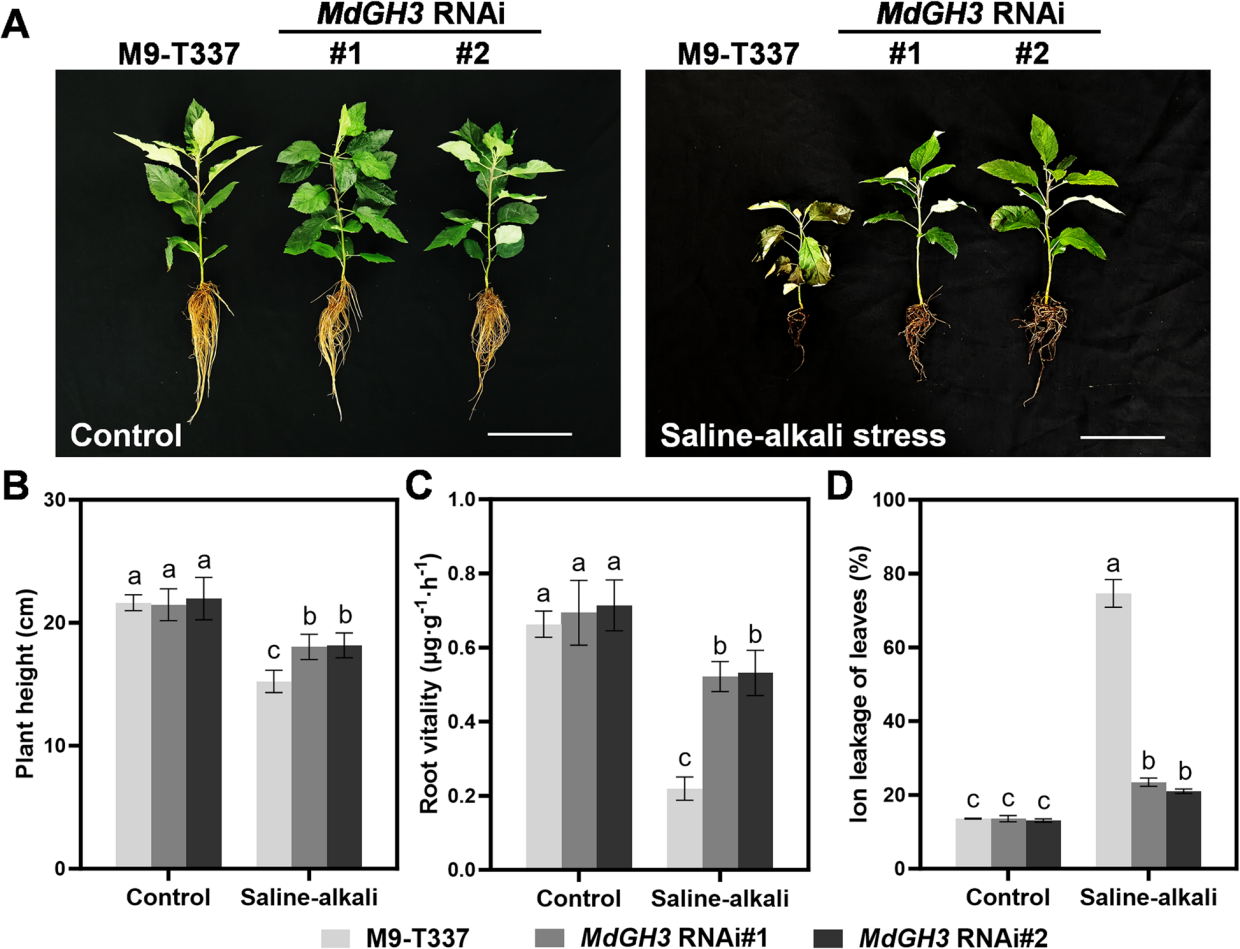
*MdGH3* RNAi plants are more tolerant to saline-alkali stress. **A** Photos of M9-T337 and *MdGH3* RNAi transgenic apple plants exposed to the control and saline-alkali stress conditions after 15 days. Scale bars: 10 cm. Plant height (**B**), root viability (**C**), ion leakage (**D**) of plants shown in a. Two-month-old M9-T337 and *MdGH3* RNAi transgenic apple plants were divided into two groups. The control group was grown in 1/2-strength Hoagland nutrient solution. The saline-alkali stress condition group was treated with 25 mM NaCl and NaHCO_3_ (v/v = 1:1, pH = 8.5 ± 0.2). After 15 days of stress treatment, plants were photographed and used in the subsequent experiments. Error bars indicate SD (*n* = 3). Statistical significance was determined by ordinary one-way ANOVA with Tukey’s multiple comparisons test

We next examined the performance of the *MdGH3* RNAi transgenic apple plants in the saline-alkali stress conditions. Two-month-old M9-T337 and *MdGH3* RNAi transgenic apple plants were divided into two groups. The control group grew in 1/2-strength Hoagland nutrient solution. The saline-alkali stress group received 25 mM NaCl and NaHCO_3_ (v/v = 1:1, pH = 8.5 ± 0.2). As shown in Fig. 1A, the plant height of *MdGH3* RNAi transgenic seedlings was comparable to that of wild-type M9-T337 under normal conditions. After a 15-day saline-alkali treatment, the *MdGH3* RNAi transgenic seedlings demonstrated much more vigorous growth potential than M9-T337. Under saline-alkali conditions, the plant height of M9-T337 was reduced by 30% compared to the control, whereas the inhibition rate of *MdGH3* RNAi transgenic seedlings was only 15–17% (Fig. 1B). Additionally, M9-T337 showed smaller, yellowing, and wilting leaves, as well as shortened and browning roots compared to the *MdGH3* RNAi plants under saline-alkali conditions. Specifically, after saline-alkali treatment, M9-T337 exhibited reductions of 32% in root length, 22% in average root diameter, and 33% in root surface area. In contrast, *MdGH3* RNAi plants showed decreases of 10–12% in root length, 19–23% in average root diameter, and 7% in root surface area (Table 1), indicating that the root system of *MdGH3* RNAi plants was significantly more developed than that of M9-T337 under saline-alkali conditions. Similarly, compared to the control, the root vitality of M9-T337 decreased by 67% after treatment, whereas that of *MdGH3* RNAi decreased only by 25–26% (Fig. 1C). Moreover, after saline-alkali treatment, the electrolyte leakage of M9-T337 was significantly higher than the control, whereas *MdGH3* RNAi exhibited only a slight increase (Fig. 1D). Overall, these results indicate that *MdGH3* RNAi plants exhibit better morphology under saline-alkali conditions compared to M9-T337.

**Table 1:** The root morphotypes of M9-T337 and *MdGH3* RNAi transgenic apple plants under control and saline-alkali stress conditions, including root length, root average diameter, and root surface area. Error bars indicate SD (*n* = 3). Statistical significance was determined by ordinary one-way ANOVA with Tukey’s multiple comparisons test

	Genotypes	Root length (cm)	Root average diameter (mm)	Root surface area (cm^2^)
Control	M9-T337	727.1 ± 5.4b	0.255 ± 0.012b	135.5 ± 2.9 d
	*MdGH3* RNAi #1	791.3 ± 8.6a	0.291 ± 0.009a	156.9 ± 5.6ab
	*MdGH3* RNAi #2	806.4 ± 9.2a	0.319 ± 0.016a	161.3 ± 3.3a
Saline-alkali stress	M9-T337	490.8 ± 28.6c	0.198 ± 0.008c	90.3 ± 6.9e
	*MdGH3* RNAi #1	707.2 ± 8.0b	0.235 ± 0.018b	145.5 ± 2.3c
	*MdGH3* RNAi #2	710.0 ± 12.6b	0.245 ± 0.012b	149.6 ± 9.9bc

### *MdGH3* RNAi plants enhance apple tolerance to saline-alkali stress by reducing the Na^+^/K^+^ ratio in leaves and roots

**Fig. 2:**
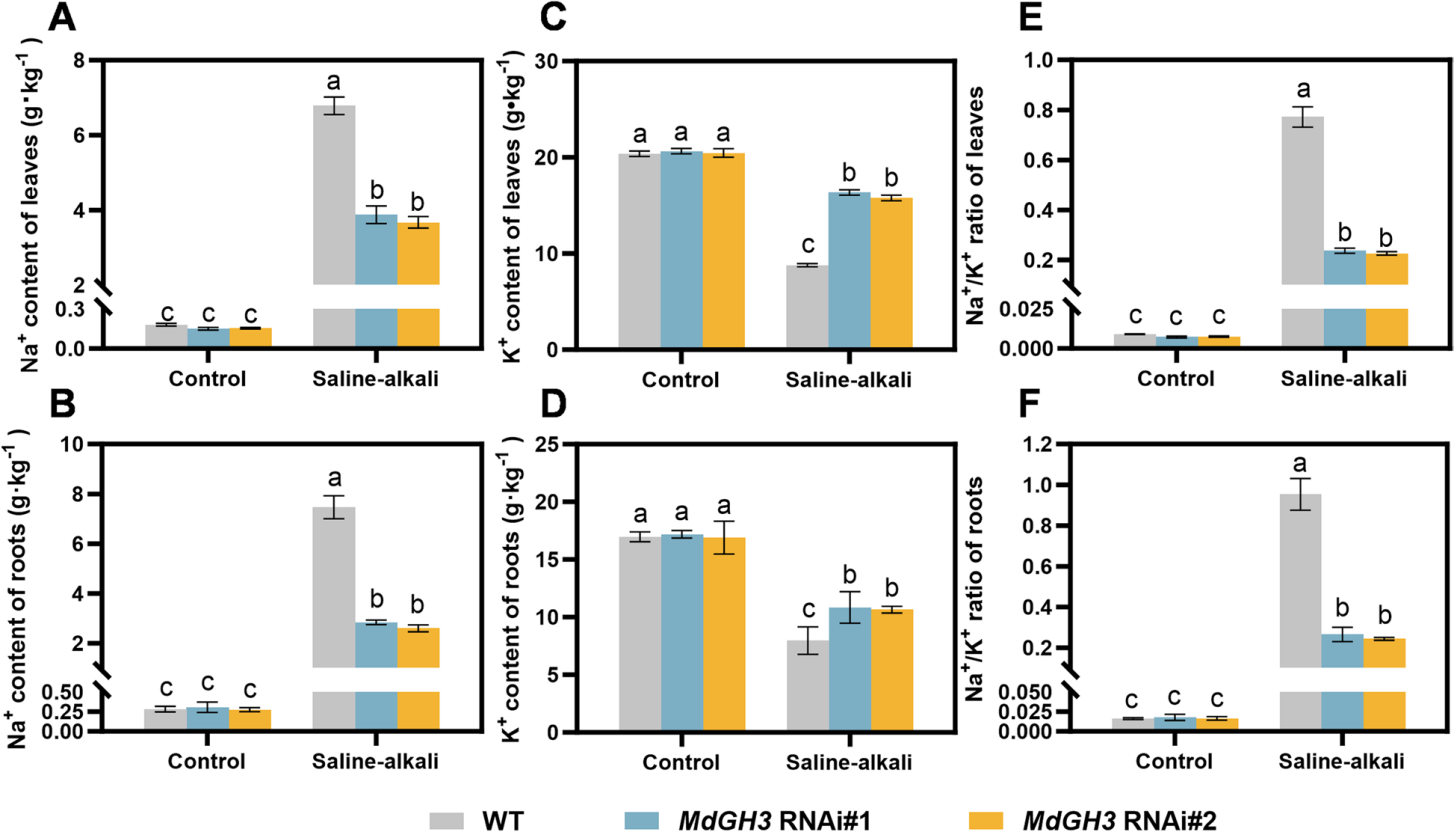
*MdGH3* RNAi plants confer saline-alkali stress tolerance by reducing the Na^+^**/**K^+^ ratio in apple leaves and roots. The Na^+^ content (**A** to **B**), K^+^ content (**C** to **D**) and Na^+^/K^+^ ratio (**E** to **F**) in the leaves and roots of M9-T337 and *MdGH3* RNAi plants under the control and saline-alkali stress conditions. Error bars indicate SD (*n* = 3). Statistical significance was determined by ordinary one-way ANOVA with Tukey’s multiple comparisons test

Saline-alkaline stress leads to an excessive concentration of Na^+^ in the soil, which enters plant cells through channels and carrier proteins, affecting the absorption of K^+^. An elevated Na^+^/K^+^ ratio can cause ionic toxicity, ultimately disrupting plant metabolism (Wang et al. 2015; Fang et al. 2021). Our results showed the low Na^+^ content in the leaves and roots of *MdGH3* RNAi and M9-T337 plants under control conditions. When exposed to the saline-alkali conditions, the Na^+^ content in the leaves and roots of all plants significantly increased. The Na^+^ content in the leaves and roots of *MdGH3* RNAi plants increased by approximately 24-fold and 8-fold, respectively, which is much lower than in M9-T337 plants (Fig. 2A and B). Under control conditions, the K^+^ content in all plant leaves and roots was about 20 g·kg^-^1. Following saline-alkali treatment, the K^+^ content in all plant leaves and roots decreased. Figure 2C and D showed that the K^+^ content in the leaves of *MdGH3* RNAi decreased by 20.8–22.9%, while approximately 56.8% in M9-T337. A similar trend was observed in the roots, with a K^+^ reduction of 35.8–36.9% in *MdGH3* RNAi plants compared to about 53.1% in M9-T337. Furthermore, the Na^+^/K^+^ ratio showed a significant change in both leaves and roots. Under saline-alkali conditions, the *MdGH3* RNAi plants had a much lower Na^+^/K^+^ ratio in leaves and roots compared to M9-T337 (Fig. 2E and F). The results suggest that *MdGH3* RNAi plants enhance apple tolerance to saline-alkali conditions by reducing the Na^+^/K^+^ ratio in leaves and roots.

### Knockdown of the* MdGH3* genes enhances the photosynthetic capacity of apple seedlings under saline-alkali stress

**Fig. 3:**
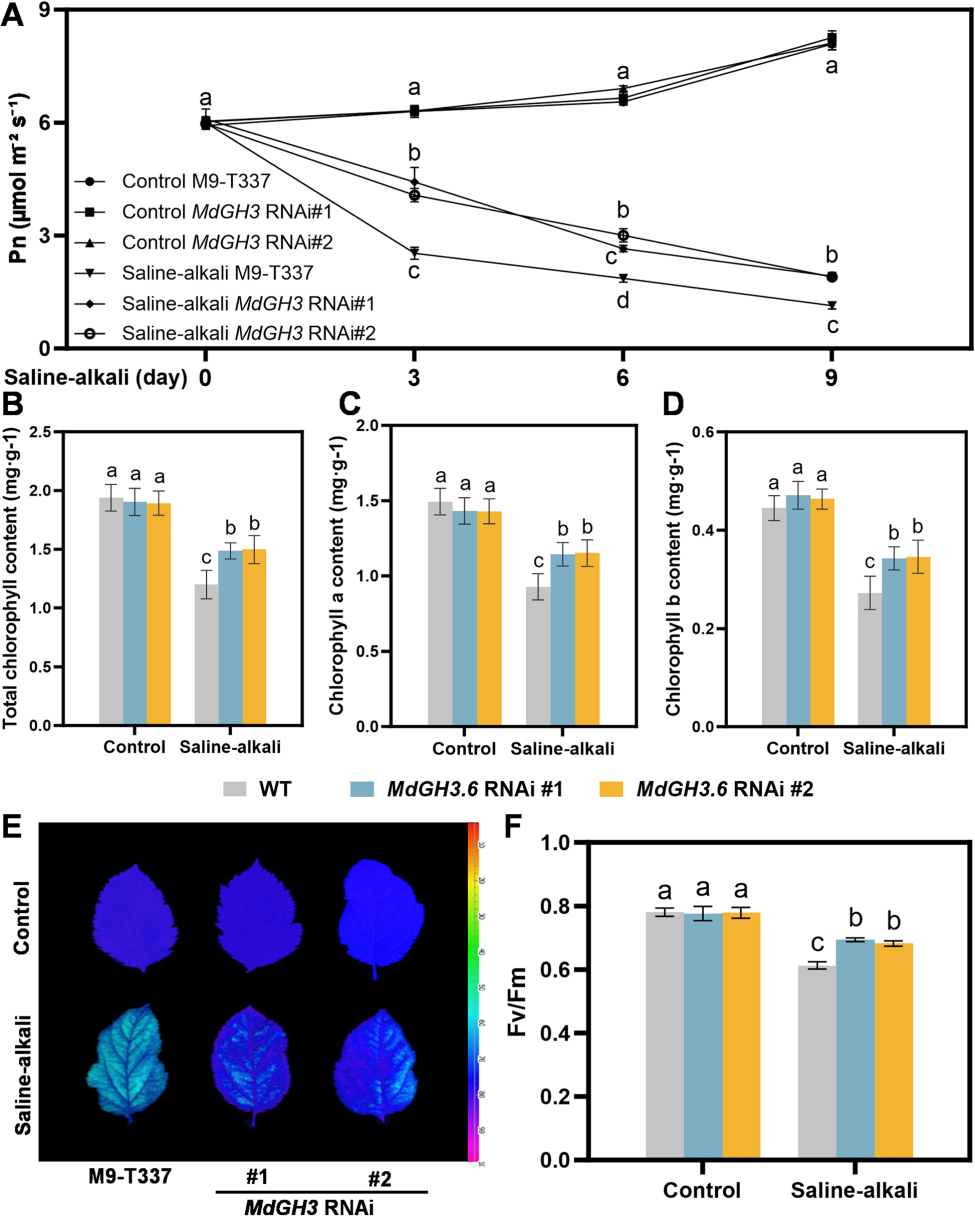
*MdGH3* RNAi plants show the enhanced photosynthetic capacity under saline-alkali stress conditions. Changes in Pn (**A**), total chlorophyll content (**B**), chlorophyll a content (**C**), chlorophyll b content (**D**), chlorophyll fluorescence (**E**), Fv/Fm (**F**) of M9-T337 and *MdGH3* RNAi transgenic apple plants under control and saline-alkali stress conditions. Error bars indicate SD (*n* = 3). Statistical significance was determined by ordinary one-way ANOVA with Tukey’s multiple comparisons test

We found the saline-alkali conditions severely inhibited the photosynthetic capacity of apple plants. As the treatment time was prolonged (0, 3, 6, 9 days), the Pn (Photosynthetic rate) of all plants decreased. However, the *MdGH3* RNAi plants maintained a higher Pn than M9-T337 under stress condition. On the 9 th day, the Pn in *MdGH3* RNAi was approximately 1.5 times higher than in M9-T337 (Fig. 3A). Chlorophyll plays a vital role in plants photosynthesis (Wang et al. 2022), thus we next detected the chlorophyll content of apple plants. The total chlorophyll content in all apple plants decreased following saline-alkali treatment, but the decline in *MdGH3* RNAi was less pronounced compared to that in M9-T337. Specifically, *MdGH3* RNAi exhibited a reduction of only 20–21% relative to the control group, while M9-T337 plants showed a decrease of 38% (Fig. 3B). Additionally, we measured the contents of chlorophyll a and chlorophyll b, both of which showed trends consistent with the total chlorophyll (Fig. 3C and D). Furthermore, the results of Fv/Fm (the maximum quantum efficiency of photosystem II photochemistry) showed that *MdGH3* RNAi decreased by approximately 10.6–12.3%, whereas it decreased about 38.1% in M9-T337 under saline-alkali treatment conditions (Fig. 3E and F). These data indicate that the knockdown of the *MdGH3* genes can mitigate the damage caused by saline-alkali stress on the photosynthetic capacity of plants, thereby enhancing plant tolerance to saline-alkali conditions.

### Knocking down *MdGH3* genes leads to enhanced antioxidant capacity under saline-alkali stress

**Fig. 4:**
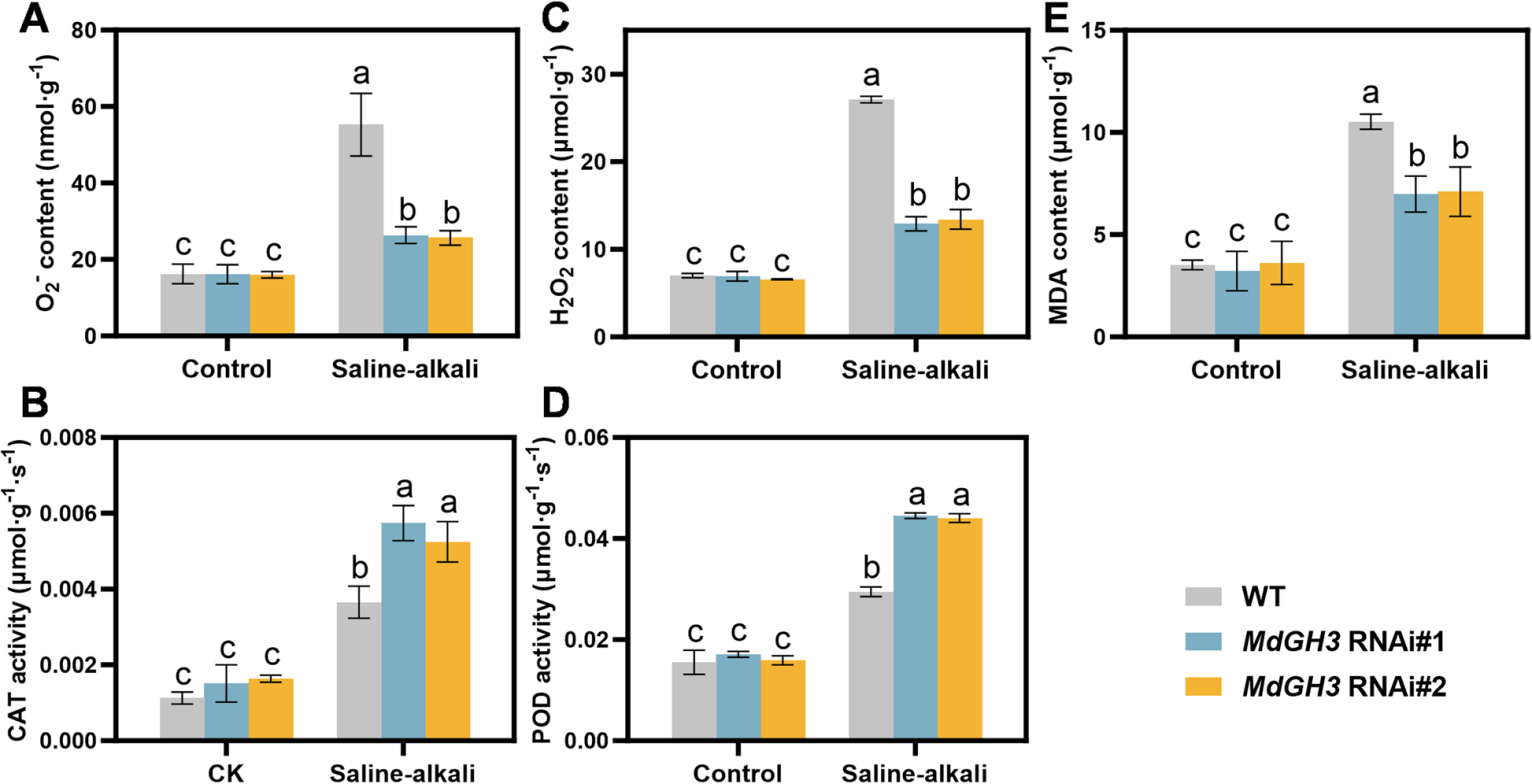
*MdGH3* RNAi plants enhance the antioxidant capacity under saline-alkali stress condition. **A** to **E** The O_2_^−^ content (**A**), H_2_O_2_ content (**B**), MDA content (**C**), activities of CAT (**D**), activities of POD (**E**) of M9-T337 and *MdGH3* RNAi plants. Two-month-old M9-T337 and *MdGH*3 RNAi transgenic apple plants were divided into two groups. The CK group grew in 1/2-strength Hoagland nutrient solution. The MSA conditions group was added with 25 mM NaCl and NaHCO_3_ (v/v = 1:1, pH = 8.5 ± 0.2). After 10 days of the stress treatment, plants were used for the subsequent experiments and analyses. Error bars indicate SD (*n* = 3). Statistical significance was determined using ordinary one-way ANOVA with Tukey’s multiple comparisons test

After 10 days of saline-alkali treatment, both M9-T337 and *MdGH3* RNAi plants accumulated more O_2_^−^ (Superoxide anion) and H_2_O_2_ (Hydrogen Peroxide) in leaves, but the increase in *MdGH3* RNAi was significantly smaller than that in M9-T337. Under saline-alkali conditions, the O_2_^−^ level in M9-T337 was 3.4 times higher than that in the control group, while the *MdGH3* RNAi was only 1.6 times (Fig. 4A). A similar trend was observed for H_2_O_2_ levels, where the increase in H_2_O_2_ for M9-T337 was 3.0–3.6 times that of *MdGH3* RNAi (Fig. 4B). MDA (Malondialdehyde) is one of the primary products of lipid peroxidation and a marker of oxidative damage to cell membranes (Jelic et al. 2021). Figure 4C showed that the MDA levels increased in both M9-T337 and *MdGH3* RNAi plants after the saline-alkali treatment. The MDA levels in M9-T337 increased about 2-fold compared to the control group, while in *MdGH3* RNAi plants, the increase was only 0.9–1.1-fold. We also checked the CAT (Catalase) and POD (Peroxidase) activities which play vital roles in scavenging ROS. After10 days of saline-alkali treatment, CAT and POD activities increased in both M9-T337 and *MdGH3* RNAi compared to the control group; however, the increase in *MdGH3* RNAi was higher than that in M9-T337 (Fig. 4D and E). These results indicate that *MdGH3* RNAi exhibits better antioxidant capacity than M9-T337 after saline-alkali treatment, enabling it to withstand saline-alkali stress better.

### Grafting GL-3 onto *MdGH3* RNAi rootstock improves apple tolerance to saline-alkali stress

**Fig. 5:**
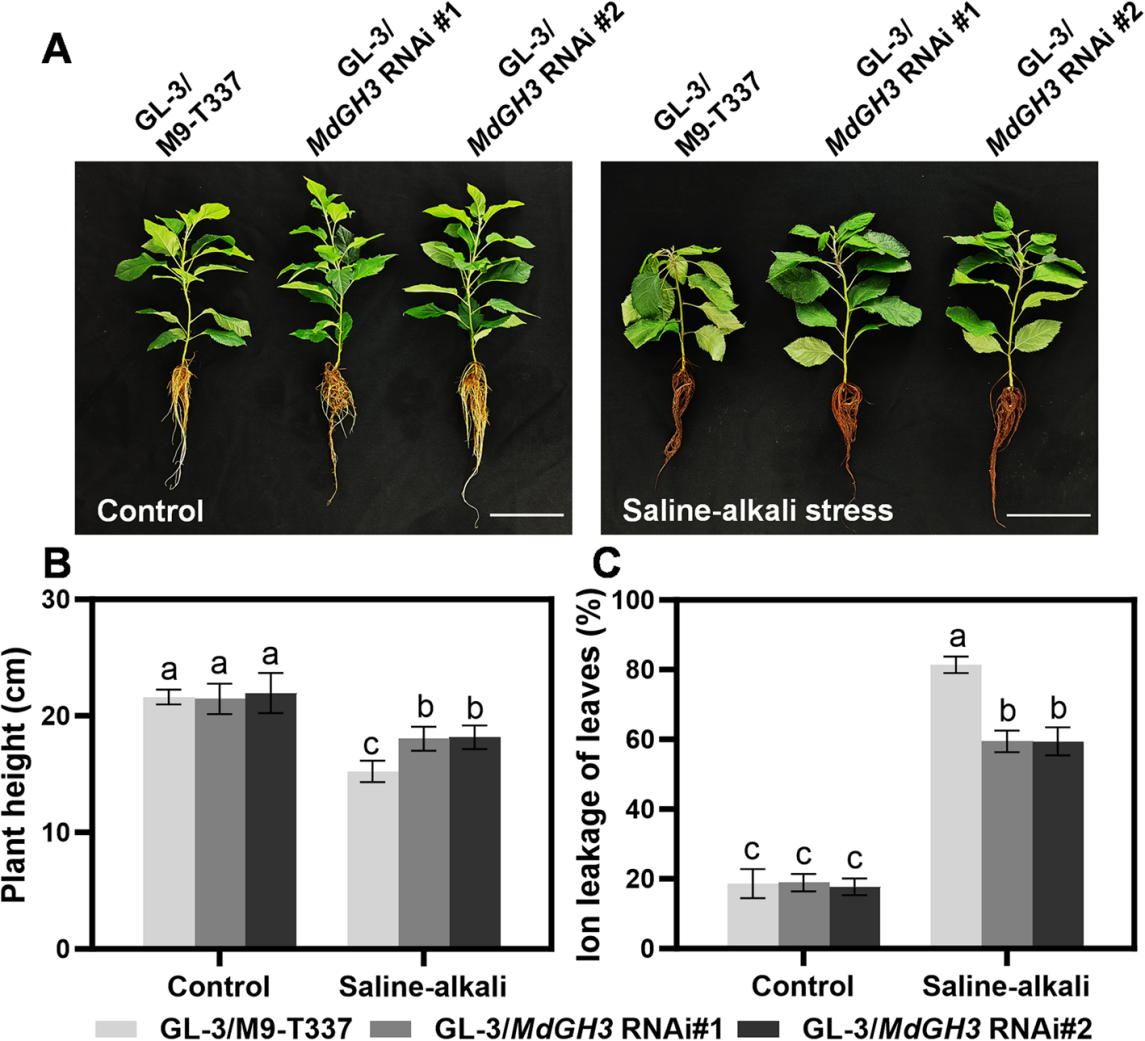
Grafting GL-3 onto *MdGH3* RNAi plants enhances the saline-alkali stress tolerance of scions. **A** Morphology of GL-3 grafted on M9-T337 and *MdGH3* RNAi plants. Scale bars: 10 cm. Plant height (**B**), ion leakage (**C**) of plants in (**A**). Two-month-old GL-3/M9-T337 and GL-3/*MdGH3* RNAi grafted plants were divided into two groups. The Control group grew in 1/2-strength Hoagland nutrient solution. The saline-alkali stress condition group was added with 25 mM NaCl and NaHCO_3_ (v/v = 1:1, pH = 8.5 ± 0.2). After 15 days of the stress treatment, plants were photographed and used in the subsequent experiments. Error bars indicate SD (*n* = 3). Statistical significance was determined by ordinary one-way ANOVA with Tukey’s multiple comparisons test

M9-T337 is one of the most widely used dwarfing rootstocks in the world; however, it has poor tolerance to abiotic stresses, especially in saline-alkali environments (Li et al. 2021). To identify the potential application of *MdGH3* RNAi as apple rootstocks, we grafted GL-3 scions onto both M9-T337 and *MdGH3* RNAi rootstocks, and then subjected these plants to saline-alkali conditions for 15 days. As shown in Fig. 5A, GL- 3/M9-T337 and GL-3/*MdGH3* RNAi exhibited similar growth vigor under control conditions. However, under saline-alkali conditions, all plants showed reduced growth rates, with smaller and yellowing leaves, shortened and browning roots. Notably, GL-3/M9-T337 was the shortest, with the most yellow leaves and the least root system compared to GL-3/*MdGH3* RNAi. Specifically, the height of GL-3/M9-T337 was 29.6% shorter than the control group, while GL-3/*MdGH3* RNAi was only 15.9–17.3% shorter (Fig. 5B). After saline-alkali treatment, the ion leakage of GL-3/M9-T337 reached to 81.3%, whereas only 56.4–58.6% in GL-3/*MdGH3* RNAi (Fig. 5C). These results indicate that the *MdGH3* RNAi rootstock can enhance the saline-alkali tolerance of scion significantly.

### Grafting GL-3 onto *MdGH3* RNAi enhances the photosynthetic capacity of apple under saline-alkali stress

**Fig. 6:**
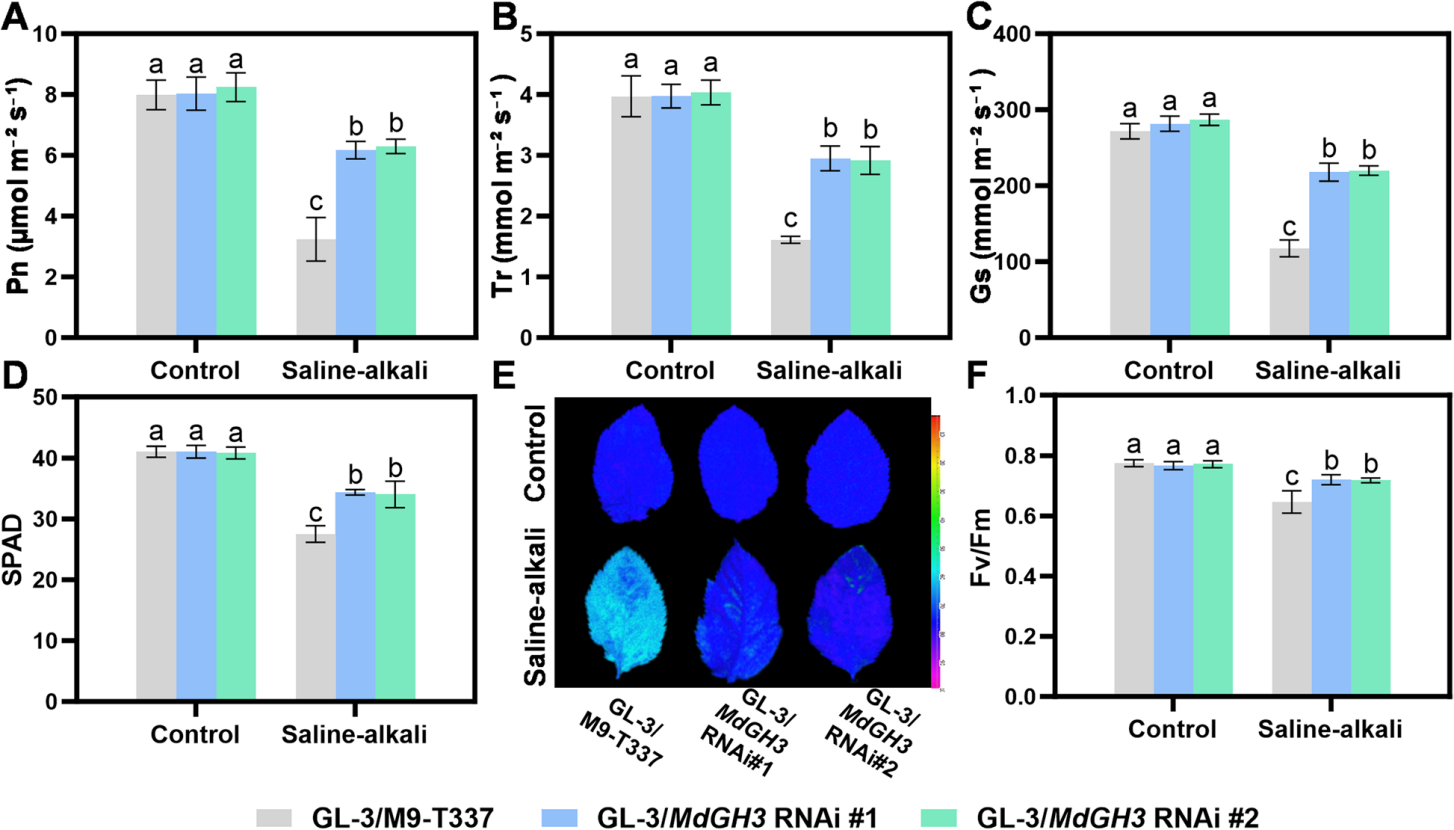
Grafting GL-3 on the *MdGH3* RNAi rootstocks facilitate the photosynthetic capacity of scions under saline-alkali stress condition. The Pn (**A**), Tr (**B**), Gs (**C**), SPAD (**D**), chlorophyll fluorescence (**E**), Fv/Fm ratios (**F**) in GL-3 grafted onto M9-T337 and *MdGH3* RNAi plants. Error bars indicate SD (*n* = 3). Statistical significance was determined by ordinary one-way ANOVA with Tukey’s multiple comparisons test

After 15 days of saline-alkali treatment, all the photosynthetic index of apple plants, including Pn, Gs (Stomatal conductance) and Tr (Transpiration rate) decreased significantly. GL-3/M9-T337 showed a 59.5% decrease in Pn, a 56.8% decrease in Gs, and a 59.4% decrease in Tr, respectively. However, these indicators in GL-3/*MdGH3* RNAi only decreased by less than 25% (Fig. 6A to C). Saline-alkali treatment also caused a reduction in SPAD (Soil and Plant Analyzer Development), which represents chlorophyll relative content. After treatment, the SPAD in GL-3/M9-T337 decreased by 32.3% compared with that in the control group. However, in GL-3/*MdGH3* RNAi, the SPAD only dropped by 16.2% to 16.6% (Fig. 6D**)**. We also measured the maximum photochemical efficiency and chlorophyll fluorescence images. The results revealed that after treatment, Fv/Fm in GL-3/M9-T337 decreased by 16.6%, while only dropped by 6.1–6.9% in GL-3/*MdGH3* RNAi (Fig. 6E and F). In summary, *MdGH3* RNAi as a rootstock can enhance the photosynthesis of scion under saline-alkali conditions.

### Grafting GL-3 onto *MdGH3* RNAi rootstocks improves antioxidant capacity under saline-alkali stress conditions

**Fig. 7:**
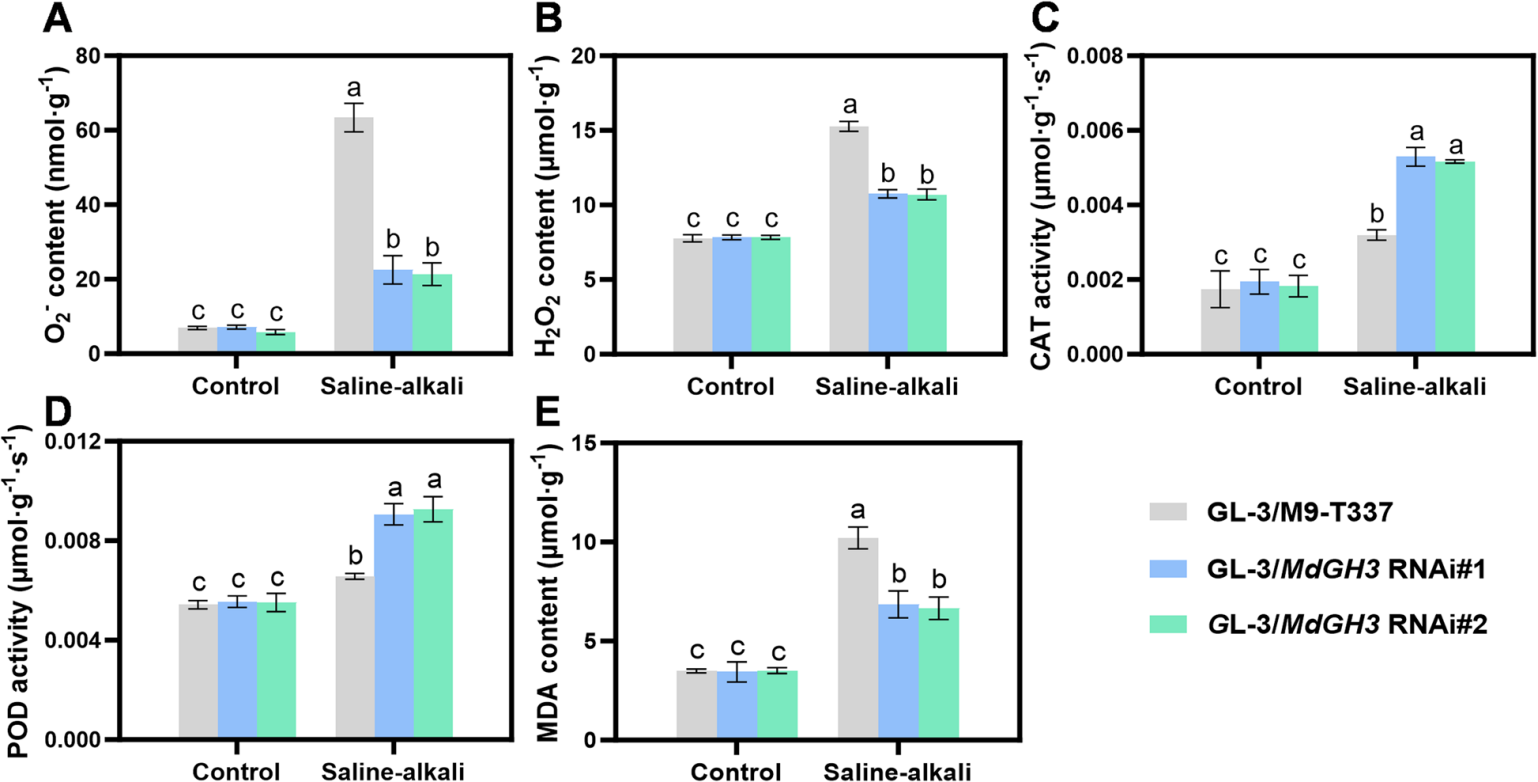
Grafting GL-3 on the *MdGH3* RNAi rootstocks confer plants tolerance to saline-alkali stress by activating antioxidant system. The O_2_^−^ content (**A**), H_2_O_2_ content (**B**), activities of CAT (**C**) and POD (**D**), MDA content (**E**) of GL-3 scions grafted onto the M9-T337 and *MdGH3* RNAi rootstocks. Error bars indicate SD (*n* = 3). Statistical significance was determined by ordinary one-way ANOVA with Tukey’s multiple comparisons test

After 10 days of saline-alkali treatment, the two main ROS components, O_2_^-^ and H_2_O_2_, significantly accumulated in all the plants. However, GL-3/*MdGH3* RNAi accumulated significantly less ROS than GL-3/M9-T337 (Fig. 7A and B). Consistently, after saline-alkali treatment, GL-3/*MdGH3* RNAi plants showed much higher CAT and POD activities compared to GL-3/M9-T337 (Fig. 7C-E). These results indicate that under saline-alkali treatment, *MdGH3* RNAi as a rootstock exhibits superior antioxidant capacity compared to M9-T337, thus enabling better resistance to saline-alkali conditions.

## Discussion

Soil saline-alkalization is becoming an increasingly serious global problem with the deterioration of natural environments. The hazards of saline-alkali soils are primarily manifested in the decline of soil quality, reduced water use efficiency, and limitations on plant growth (Guo et al. 2023; Li et al. 2010; Shi et al. 2021). Apple production is dependent on grafting mediated by the rootstocks to promote early flowering, better quality, higher yield and enhanced stress tolerance (Webster 1995; Turan & Tripathy 2015; Cimen and Yesiloglu, 2016). The widely used commercial apple rootstock M9-T337 is dwarfing, but stress sensitive (Tabakov et al. 2016; Xian et al. 2024). The urgent requirement in apple production is to enhance the tolerance of M9-T337. Our previous research found the potential application of *MdGH3* RNAi transgenic apple (in GL-3 background) as a rootstock to be more tolerant to drought stress than the wild type, and the capability of *MdGH3* RNAi rootstock can promote flowering, fruit setting and fruit size of the scion as the dwarfing rootstock M9-T337 (Jiang et al. 2022a, b). Although the *MdGH3* RNAi transgenic apple plants (in GL-3 background) showed drought tolerance, they lacked the dwarfing traits, which are the main advantage of M9-T337. To obtain a dwarfing and stress-tolerant rootstock, we first knocked down the drought-negative regulator *MdGH3.6* and its five paralogues in the background of M9-T337. The *MdGH3* RNAi transgenic apple plants showed much greater tolerance than the wild type M9-T337 under the saline-alkali stress conditions. The *MdGH3* RNAi transgenic apple seedlings actively responded to saline-alkali stress on the basis of different aspects: (1) morphological adaptations, including biomass, root system and viability (Fig. 1 and Table 1); (2) physiological adaptations, including photosynthesis activity, osmotic regulation, ion balance, and antioxidant effects (Figs. 2, 3 and 4). Previous studies found the key roles of auxin catabolism mediated by *GH3* genes in regulating plants root development. For example, Arabidopsis *GH3*-mediated auxin inactivation weakens plants lateral root development, the *gh3oct* plants with mutated Arabidopsis *gh3.1,2,3,4,5,6,9,17* had an elaborated root architecture and increased tolerance to salt and drought stresses (Casanova-Sáez et al. 2022). Overexpression of *CsGH3.4* inhibited the development of adventitious roots in tea (*Camellia sinensis*) plants (Wang et al. 2024), silencing *GH3.6* gene and its five paralogues in apple led to more developed root systems under drought conditions (Jiang et al. 2022a, b). The root system of plants is responsible for absorbing water and nutrients, and its growth directly determines the vigor of the above-ground parts of the plants. Thus, the root development mediated by *GH3.6* usually affects plants morphology in response to stress conditions. As our study showed, the more developed root system and higher viability contributed to the enhanced saline-alkali stress tolerance of *MdGH3* RNAi plants.

Under saline-alkali stress conditions, excessive Na^+^ in the rhizosphere inhibits the uptake of K^+^, a critical mineral for plant metabolism that plays a role in photosynthesis, carbohydrate transport, and protein synthesis. A high Na^+^/K^+^ ratio damages plant structures and harms growth (Fang et al. 2021; Sun et al. 2021). In our study, the Na^+^/K^+^ ratio in *MdGH3* RNAi plants was lower than that in M9-T337 under the saline-alkali conditions (Fig. 2E and E), indicating that the *MdGH3* RNAi plants experienced less damage from saline-alkali stress. Additionally, in saline environments, high concentrations of Na^+^, chloride ions (Cl^-^), sulfate ions (SO_4_^2^^-^), and bicarbonate ions (HCO_3_^-^) significantly inhibit the plants’ absorption and utilization of elements like K^+^, phosphorus (P), and magnesium (Mg). These elements, particularly N, P, and Mg, are crucial components of chloroplasts, and their deficiency can damage chloroplast structure, leading to decreased chlorophyll content and subsequently reduced photosynthesis (Turan & Tripathy 2015; Tsai et al. 2019). Silencing *GH3.5* reduced chlorophyll content in cotton under drought stress, leading to decreased photosynthesis. Our research showed that *MdGH3* RNAi plants had more total chlorophyll content, higher Pn, and Fv/Fm than M9-T337 under saline-alkali stress conditions, which might be due to the lower Na^+^/K^+^ ratio in the *MdGH3* RNAi plants.

Under saline-alkali stress, plant cells experience oxidative stress due to high salinity and pH, leading to elevated levels of reactive ROS. When ROS accumulated excessively, it causes oxidative damage to cells, impairing plant growth and survival. Plants usually activate their antioxidant defense systems to eliminate excess ROS, including CAT and POD (Guo et al. 2023). In our study, ROS accumulation in *MdGH3* RNAi plants was lower than that in M9-T337 under stress conditions. Consistently, CAT and POD activities were higher than those in M9-T337, indicating that *MdGH3* RNAi has a more robust antioxidant system (Fig. 4). This result is consistent with Jiang et al. (2022a, b), who demonstrated that knocking down *MdGH3* in GL-3 apple reduced H_2_O_2_ content by activating antioxidant enzyme activity under drought stress (Jiang et al. 2022b). The primary function of GH3 genes is to catalyze the conjugation of IAA with amino acids. Exogenous IAA can enhance the activity of antioxidant enzymes of mango (*Mangifera indica*) fruit to delay cell wall metabolism after harvest (Zhou et al. 2023). We hypothesize that the IAA balance mediated by *MdGH3* in response to saline-alkali conditions might contribute to the stress tolerance of *MdGH3* RNAi plants.

Studies have shown that using salt-tolerant rootstocks can enhance the yield of pepper (*Capsicum annuum*) scions under saline-alkali stress by maintaining its photosynthetic capacity and sink strength (Padilla et al. 2024). Additionally, the saline-alkali-tolerant rootstocks can enhance the drought tolerance of scions by modifying antioxidant enzyme activities, increasing photosynthetic capacity, and reducing ROS accumulation under drought stress (Zhang et al. 2019; Shehata et al. 2022). In our study, we engineered M9-T337, a widely used dwarfing rootstock, with enhanced tolerance to saline-alkaline stress by knocking down *MdGH3.6* and its five paralogues. As rootstocks, the *MdGH3* RNAi plants exhibited similar height compared to M9-T337 under control conditions, indicating the potential of its application as a dwarfing apple rootstock. Additionally, using *MdGH3* RNAi as a rootstock significantly improved the performance of the scion under saline-alkali stress, including stronger antioxidant capacity and higher photosynthetic performance (Figs. 5, 6 and 7). The improvement is partly attributed to the more robust root system of *MdGH3* RNAi plants under saline-alkali stress. This suggests the application potential of *MdGH3* RNAi plants as a dwarfing and stress-tolerant apple rootstock.

## Conclusion

In summary, we used RNAi technology to knock down *GH3* genes and its five paralogues in M9-T337 to engineer a dwarfing and saline-alkali-tolerant rootstock. The *MdGH3* RNAi transgenic plants exhibited more robust root systems, lower Na^+^/K^+^ ratio, enhanced photosynthetic capability, and stronger antioxidant system than M9-T337 under saline-alkali conditions. When used as rootstocks, *MdGH3* RNAi plants improved the scion’s plant height, photosynthetic efficiency and antioxidant capacity in saline-alkali stress conditions, indicating the potential of *MdGH3* as a dwarfing and stress-tolerant apple rootstock.

## Materials and methods

### Plant material and growth conditions

Tissue-cultured M9-T337 (used as the background for apple transformation), transgenic plants, and grafted plants were sub-cultured every 4 weeks on Murashige & Skoog (MS) medium (4.43 g/L MS salts, 30 g/L sucrose, 0.1 mg/L 6-Benzylaminopurine [6-BA],0.2 mg/L 3-Indoleacetic acid [IAA], and 7.5 g/L agar, pH 6.0) under long-day conditions (14 h light [cool white, 100 µmol m^−2^ s^−1^, T5 LED batten]: 10 h dark) at 25℃ (Niu et al. 2022). Tissue-cultured plants were rooted, transplanted into soil, and grown in a growth chamber at Northwest A&F University, Yangling, China (34°200’ N, 108°240’ E) (16-h light:8-h dark, 25℃, 55% relative humidity). After transplanting into soil for 2 weeks, all the plants were ready for stress treatment.

### Genetic transformation

The *MdGH3* RNAi vector was previously constructed by Jiang (Jiang et al. 2022a, b). We transformed the vector into *Agrobacterium tumefaciens* and infected the tissue-cultured wild-type, M9-T337. The transgenic plants were screened on MS medium (4.43 g/L MS salts, 30 g/L sucrose, 0.1 mg/L 6-BA, 0.2 mg/L IAA, and 7.5 g/L agar, pH 6.0) supplemented with 50 mg/L kanamycin and 250 mg/L cefotaxime (Li et al. 2022). The transgenic plants were then verified in DNA and RNA levels (Guo et al. 2024).

### Micrografting of tissue-cultured seedlings

Under sterile conditions, 40-day-old tissue-cultured seedlings used as rootstocks were topped, leaving a stem segment approximately 1.5 cm in length. The bottom lateral bud was removed, and a longitudinal incision approximately 0.5 cm in length was made at the top of the rootstock. A scion of similar thickness to the rootstock and approximately 1.5 cm in length was selected, retaining the top 2 to 4 leaves. The base of the scion was cut into a wedge shape (approximately 0.5 cm in length) and inserted into the rootstock. The graft interface was securely wrapped with tin-platinum paper. Finally, the grafted seedlings were transferred to culture medium for further growth.

### Saline-alkali stress treatment

The two-month-old plants grew in soil were transferred to a hydroponic system for saline-alkali stress treatment. The hydroponic containers measured 30 × 21 × 9 cm3 and were filled with 3.5 L of half-strength Hoagland nutrient solution. The growth conditions were as follows: a 14-h photoperiod (light intensity of 160 μmol m^-^2∙s^-^1), day/night temperatures of 25 ± 2 °C/18 ± 2 °C, and a relative humidity of 65 ± 5%. The seedlings were divided into two groups: (1) Control, half-strength Hoagland nutrient solution; (2) Saline-alkali stress conditions, half-strength Hoagland nutrient solution containing 25 mM NaCl and 25 mM NaHCO_3_ (v/v = 1:1), with a pH of 8.5 ± 0.2. The treatment lasted for 15 days (Fan et al. 2024).

### Total RNA extraction and qRT-PCR analysis

The CTAB method was used to extract the total RNA from apple leaves (Chang et al. 1993) and the endogenous DNA was removed by RNase-free Dnase I (Thermo Scientific, EN0521, USA). Three biological replicates were used for detection. For RT-qPCR analysis, first strand cDNA was synthesized according to the manufacturer’s instructions of the RevertAid First Strand cDNA Synthesis Kit (Thermo Scientific, USA). The RT-qPCR was performed in a total 20 µL reaction containing GoTaq qPCR Master Mix (Promega, USA). The primers used are listed in Table S1.

### Plants morphology analysis

Plant height, fresh weight and dry weight were measured directly after 15-day-treatment. Total root length, root surface area, root volume, and average diameter were measured using root scanner (Epson Expression 10000XL, Japan). Root material was collected after the 15-day-treatment and the root viability was assessed according to the instructions provided with the reagent kit (Solarbio BC5270, China).

### Measurement of electrolyte leakage

The mature leaves from the middle of the plants were collected for electrolyte leakage detection. Leaf disc samples were placed in a 15 mL clean tube containing 8 mL of deionized water and soaked at room temperature for 4 h. The conductivity of the solution (C_1_) was measured by conductivity meter (Leici DDS- 307). Afterwards, the samples were boiled for 20 min, and the conductivity (C_2_) was measured once the tubes had cooled to room temperature. The ion leakage was calculated as C_1_/C_2_.

### O_2_^−^, H_2_O_2_, MDA and antioxidant enzyme activity measurements

O^2−^ content was measured using a Hydrogen Peroxide Assay Kit (Comin, SA-2-G, China). H_2_O_2_ content was measured using a Hydrogen Peroxide Assay Kit (Comin, H2O2-1-Y, China). The activities of POD and CAT were measured as described previously (Wang et al. 2012). MDA content was determined according to the methods described previously (Sun et al. 2018).

### Measurement of photosynthetic parameters

The LI-COR 6800 portable photosynthetic (LI-COR 6800, Lincoln, NE, USA) system was used to determine photosynthetic parameters. The mature fifth to sixth leaves from the apical bud were measured between 09:00 and 11:00 a.m. The Pn, Gs, and Tr of each leaf were measured using LED red and blue light source with 1,000 μmol m^−2^s^−1^ light intensity and 500 μmol s^−1^air flow. SPAD measurements were taken with SPAD-502 (Konica Minolta, Japan). When detecting the chlorophyll fluorescence (Fv/Fm), the fully expanded functional leaves were firstly wrapped in aluminum foil for a 20-min-dark adaptation, and then placed in a chlorophyll fluorescence imaging system (HEXAGON-IMAGING-PAM, Heinz Walz, Germany) for analysis. Three biological replicates were conducted.

### Determination of Na^+^ and K^+^ ion content

Mature leaves were randomly selected for the determination of Na^+^ and K^+^ ion content. The leaf material was dried at 105 °C for 20 min, then transferred to 65 °C for at least 72 h. The completely dried leaves were grinder into a fine powder for analysis. The Na^+^ and K^+^ ion contents were determined by flame photometry (M410; Sherwood Scientific, Cambridge, UK).

### Statistical analysis

Data are presented as the mean ± standard deviation. Statistical significance was determined by ordinary one-way ANOVA with Tukey’s multiple comparisons test using SPSS (version 21.0).

## Supplementary Information

Supplementary Material 1: Fig. S1. Identification of *MdGH3* RNAi transgenic apple plants. (A) DNA level identification of *MdGH3* RNAi transgenic plants. mRNA level of *MdGH3.6* (B), *MdGH3.6-like* (C), *MdGH3.1-1* (D), *MdGH3.1-2* (E), *MdGH3.1-3* (F), *MdGH3.1-4* (G) in M9-T337 and *MdGH3* RNAi transgenic apple seedlings. Error bars indicate SD (*n* = 3). Statistical significance was determined by ordinary one-way ANOVA with Tukey’s multiple comparisons test. M2000, DNA Marker 2000. The positive control used MdGH3-pHELLS-GATE2 as the template. Table S1. Primers used in the present study.

## Data Availability

All data generated or analyzed during this study are included in this published article.

## Abbreviations

RNAi: RNA interference
ROS: Reactive Oxygen Species
MDA: Malondialdehyde
GMG: Enetically modified
IAA: Indole-3-acetic acid
O_2_^−^: Superoxide anion
H_2_O_2_: Hydrogen Peroxide
Pn: Photosynthetic rate
CAT: Catalase
POD: Peroxidase
Gs: Stomatal conductance
Tr: Transpiration rate
PSII: Photosystem II
SPAD: Soil and Plant Analyzer Development
Fv/Fm: The maximum quantum efficiency of photosystem II photochemistry
qRT-PCR: Real-time quantitative PCR
