# Supplementary figures and images for: Engineering saline-alkali-tolerant apple rootstock by knocking down MdGH3 genes in M9-T337
Source: Stress Biol. 2025 Jun 23;5(1):44. doi: 10.1007/s44154-025-00236-7 (PMC12185813; doi:10.1007/s44154-025-00236-7)

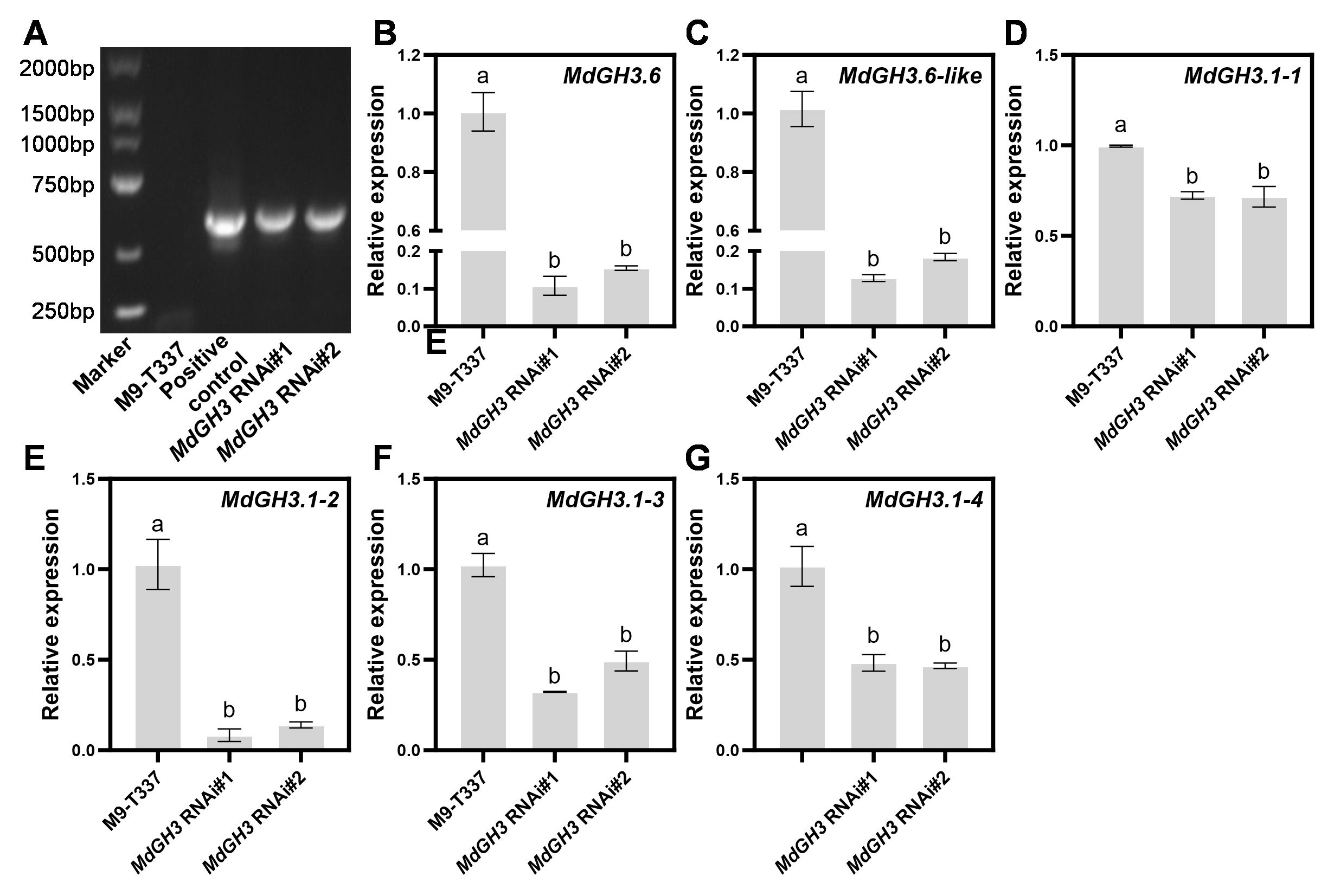

Supplement: Supplementary file 1 — Supplementary Material 1: Fig. S1. Identification of MdGH3 RNAi transgenic apple plants. (A) DNA level identification of MdGH3 RNAi transgenic plants. mRNA level of MdGH3.6 (B), MdGH3.6-like (C), MdGH3.1-1 (D), MdGH3.1-2 (E), MdGH3.1-3 (F), MdGH3.1-4 (G) in M9-T337 and MdGH3 RNAi transgenic apple seedlings. Error bars indicate SD (n = 3). Statistical significance was determined by ordinary one-way ANOVA with Tukey’s multiple comparisons test. M2000, DNA Marker 2000. The positive control used MdGH3-pHELLS-GATE2 as the template. Table S1. Primers used in the present study. [file 44154_2025_236_MOESM1_ESM.tif]
